# Study design of the DAS-OLT trial: a randomized controlled trial to evaluate the impact of dexmedetomidine on early allograft dysfunction following liver transplantation

**DOI:** 10.1186/s13063-020-04497-7

**Published:** 2020-06-26

**Authors:** Chenlu Ni, Joe Masters, Ling Zhu, Weifeng Yu, Yingfu Jiao, Yuting Yang, Cui Cui, Suqing Yin, Liqun Yang, Bo Qi, Daqing Ma

**Affiliations:** 1 Department of Anesthesiology, Ren Ji Hospital, Shanghai Jiao Tong University School of Medicine, No. 160 Pujian Road, Shanghai, 200127 China; 2 Anaesthetics, Pain Medicine and Intensive Care, Department of Surgery and Cancer, Faculty of Medicine, Imperial College London, Chelsea and Westminster Hospital, London, UK; 3 Department of Anesthesiology, Ren Ji Hospital, Shanghai Jiao Tong University School of Medicine, Shanghai, 200136 China

**Keywords:** Liver transplantation, Dexmedetomidine, Early allograft dysfunction, Primary graft dysfunction

## Abstract

**Background:**

Perioperative ischemia/reperfusion (I/R) injury during liver transplantation is strongly associated with early allograft dysfunction (EAD), graft loss, and mortality. Hepatic I/R injury also causes remote damage to other organs including the renal and pulmonary systems. Dexmedetomidine (DEX), a selective α2-adrenoceptor agonist which is used as an adjuvant to general anesthesia, has been shown in preclinical studies to provide organ protection by ameliorating the effects of I/R injury in a range of tissues (including the liver). However, prospective clinical evidence of any potential benefits in improving outcomes in liver transplantation is lacking. This study aimed to verify the hypothesis that the application of dexmedetomidine during the perioperative period of liver transplantation can reduce the incidence of EAD and primary graft non-function (PNF). At the same time, the effects of dexmedetomidine application on perioperative renal function and lung function were studied.

**Methods:**

This is a prospective, single-center, randomized, parallel-group study. Two hundred participants (18–65 years) scheduled to undergo liver transplantation under general anesthesia will be included in this study. For participants in the treatment group, a loading dose of DEX will be given after induction of anesthesia (1 μg/kg over 10 min) followed by a continuous infusion (0.5 μg/kg /h) until the end of surgery. For participants in the placebo group, an equal volume loading dose of 0.9% saline will be given after the induction of anesthesia followed by an equal volume continuous infusion until the end of surgery. All other supplements, e.g., opioids, sedatives, and muscle relaxant, will be identical in both arms and administered according to routine clinical practice.

**Discussion:**

The present trial will examine whether DEX confers organoprotective effects in the liver, in terms of reducing the incidence of EAD and PNF in orthotopic liver transplantation recipients.

**Trial registration:**

ClinicalTrials.gov NCT03770130. Registered on 10 December 2018. https://clinicaltrials.gov/ct2/show/NCT03770130

## Introduction

Hepatic failure is associated with considerable morbidity and mortality. Decompensated cirrhosis with the development of ascites has a 2-year survival rate of only around 50% [1]. Cirrhosis affected an estimated 2.8 million people worldwide and resulted in 1.8 million deaths in 2015 [2, 3]. Hepatic failure also has multiorgan implications with detrimental effects on cardiovascular, pulmonary, renal, and neurological systems. Liver transplantation (LT), being the most effective treatment for patients with end-stage liver disease, confers a significant survival benefit (85–95% 1-year survival in many centers; 70–80% alive at 5 years) [4, 5].

### Early allograft dysfunction (EAD) and primary non-function (PNF)

Due to a widespread shortage of donor organs, there has been an increase in the acceptance of organs of marginal quality that are particularly prone to perioperative injury and are at increased risk of early postoperative dysfunction and failure [6, 7]. There is therefore significant interest in the development of new strategies to prevent liver graft dysfunction and early graft loss. Primary non-function (PNF) occurs in up to 7% of grafts and requires urgent retransplantation [4]. Initial dysfunction of the liver graft (EAD or PNF) in the immediate postoperative period is indicative of hepatocellular damage and synthetic impairment and is strongly predictive of poor graft and patient survival [8–11]. As such, EAD serves as an important surrogate end point in LT clinical trials.

The incidence of EAD is approximately 25% in most large studies, although reports range from 9.3 to 43.7% [4, 9, 12, 13]. EAD is multifactorial; among other factors, donor and recipient characteristics play a role. Currently, no treatment to prevent EAD is available.

### Choice of anesthetic drugs for liver transplantation

Anesthesia for LT has evolved significantly since the first transplants in the 1960s, with increasing recognition that the anesthetist plays a key role in influencing outcomes (such as graft function and survival) by optimizing parameters such as the patient’s hemodynamic status, coagulation profile, and volume status. In addition to this, it has been postulated that the type of anesthetic drugs administered in the perioperative period may also affect outcomes [14, 15].

Hepatic ischemia/reperfusion (I/R) injury is a recognized complication of LT and is strongly linked to EAD, PNF, longer hospital stays, and lower long-term graft survival [14]. Therefore, strategies to attenuate I/R injury may improve graft function and survival.

Dexmedetomidine (DEX), a selective α2-adrenoceptor agonist, is a useful adjuvant to general anesthesia which has sedative, anxiolytic, analgesic, and hypotensive properties. In vitro and in vivo preclinical studies have widely demonstrated that DEX provides organ protection in kidney, lung, brain, heart, and liver tissues by ameliorating I/R injury, inhibiting pro-inflammatory signaling pathways, and decreasing cell death [16–25].

Regarding hepatic protection, both Chen et al. and Wang et al. have demonstrated that DEX treatment protects against liver I/R injury in rodents. Inhibition of the toll like receptor 4 (TLR4)/nuclear factor kappa B (NF-κB) pathway has been implicated as one of the mechanisms underlying these protective effects [26, 27]. In the clinical setting, a randomized controlled trial by Wang et al. of 44 patients undergoing hepatectomy found that intraoperative treatment with DEX (loading dose of 1 μg/kg over 10 min followed by a maintenance dose of 0.3 μg/kg/h) resulted in lower serum AST and ALT levels in the first 72 h postoperatively. However, the trial only looked at these biomarkers of hepatocellular damage and did not study patient outcomes [28]. Likewise, a study by Fayed et al. of 40 patients undergoing live donor LT showed that intraoperative infusion of DEX (0.8 μg/kg/h) improved postoperative liver function tests (on days 1, 3, and 5) and showed improved histopathological changes in liver tissue taken by biopsy at the end of surgery compared to controls. Again the authors did not look at long-term graft function or survival, and it is unclear whether these initial biochemical and histological benefits were associated with an improved clinical picture and better patient prognosis [29].

Considering the strength of these preclinical data demonstrating the organoprotective effects of DEX, urgent randomized clinical trials are needed to evaluate its potentially beneficial impact in LT. This study aimed to verify the hypothesis that the application of dexmedetomidine during the perioperative period of liver transplantation can reduce the incidence of EAD and primary graft non-function (PNF). At the same time, the effects of dexmedetomidine application on perioperative renal function and lung function were studied.

### Effects of dexmedetomidine on dysfunction of other organs following liver transplantation

#### Renal function

Acute kidney injury (AKI) occurs in 12–94% of patients undergoing LT (depending on the definition of AKI used) and is associated with increased morbidity and mortality [30, 31]. The etiology is multifactorial and is likely to involve renal I/R injury following renal hypoperfusion as a result of perioperative hypotension, intestinal endotoxemia induced by inferior vena cava (IVC) occlusion during surgery, and remote kidney damage induced by hepatic I/R injury [32, 33]. EAD has been shown to be an independent risk factor for AKI [34]. Perioperative interventions to reduce incidence are currently limited to supportive therapies and include volume expansion and vasopressors to optimize renal perfusion. Early renal replacement therapy is indicated if these measures fail with 4.5–21% of LT patients requiring support [31]. Other pharmacological interventions such as dopamine, mannitol, and diuretics have not been shown to have any benefit.

Preclinical studies have widely reported the renoprotective effects of DEX following renal ischemia-reperfusion [18, 35–38]. In the context of LT, both Yu et al. and Yao et al. have demonstrated that pre-treatment with DEX reduces AKI following orthotopic autologous LT in rats by suppressing TLR4/NF-κB pathway activation [22] and by enhancing antioxidant levels through activation of nuclear factor erythroid 2-related factor 2 [23].

#### Pulmonary function

Acute respiratory distress syndrome (ARDS) is a common complication following LT with an incidence up to 42% [39]. ARDS is one of the major causes of death following LT. Hepatic I/R injury, and the subsequent systemic release of inflammatory mediators has again been implicated in the etiology of postoperative pulmonary injury [40].

A number of studies have demonstrated that DEX has cytoprotective effects in the lungs: Cui et al. have shown that DEX protects against bilirubin-induced lung alveolar epithelial cell death in vitro and in vivo [20]. Gu et al. reported that DEX attenuates remote lung injury induced by renal I/R injury in mice [24]. And, most relevant to this study, Chi et al. have shown that DEX ameliorates lung injury following orthotopic autologous LT in rats by inhibiting the TLR4/NF-κB pathway [25].

Given these preclinical findings, it is important that the impact of DEX on postoperative renal and pulmonary function in patients undergoing LT be studied in clinical trials.

## Methods

### Study settings

DSA-OLT is a single-center, randomized, double-blind, placebo-controlled, parallel-group study. We plan to enroll 200 adult participants scheduled for liver transplantation surgery to evaluate the impact of dexmedetomidine on allograft function and survival following cadaveric liver transplantation. This trial is registered at www.clinicalTrials.gov with a registration number of NCT03770130. The primary endpoint is the incidence of EAD following surgery.

### Study population

#### Inclusion criteria

Participants will be considered for inclusion if they meet the following criteria:

1. Age ≥ 18 years and < 65
2. Scheduled to undergo allogenic liver transplant surgery under general anesthesia
3. Participants should meet the UCSF criteria [41] (single tumor diameter ≤ 6.5 cm; multiple tumors ≤ 3, maximum diameter ≤ 4.5 cm; cumulative diameter ≤ 8 cm; without large vessel infiltration and extrahepatic metastasis)
4. Agree to participate and give written informed consent

#### Exclusion criteria

1. Severe renal dysfunction (undergoing renal replacement therapy before surgery)
2. Severe pulmonary dysfunction (severe pre-existing chronic lung disease)
3. Severe circulatory instability (severe coronary artery disease, unstable angina, left ventricular ejection fraction < 30%, sick sinus syndrome, severe sinus bradycardia [< 50 bpm], second-degree or greater atrioventricular block)
4. Known allergy or intolerance to trial medication
5. Participation in other clinical trials within 30 days prior to randomization
6. Retransplantation
7. Multiple organ transplantation
8. Other reasons that are considered unsuitable for study participation by the responsible surgeon or anesthetist (reasons must be documented in the case report form [CRF])

#### Drop out criteria

1. Withdrawal of consent by the participants or their legal representative
2. Loss to follow-up

For drop out cases, the detailed reason will be recorded and the primary therapeutic effects last recorded will be regarded as the final results. The CRFs of these cases will be preserved for future reference.

#### Rejection criteria

Enrolled cases who meet any of the following criteria will be excluded from per protocol analysis:

1. Anesthesia not performed according to the protocol
2. No study record

For rejected cases, the detailed reasons will be documented. However, subsequent study procedure will be performed according to the protocol and the CRFs will be preserved for future reference. These cases will be included in the intention-to-treat analysis.

#### Termination criteria

The trial will be suspended if:

1. Serious safety issues occur during the study
2. Serious errors are found in the study protocol
3. Funding or management issues for the researchers occur
4. The study is canceled by executive

Study termination may be short-lived or permanent. If the study is terminated early, a written report should be submitted to the Ethics Committee. All CRFs and recorded files should be kept for reference. The resumption of research will require the approval of an ethics committee.

### Purposes of the study

#### Primary outcomes

Incidence of EAD following surgery

- Defined according to Olthoff’s criteria published in 2010: (1) bilirubin ≥10 mg/dL on day 7; or (2) INR > 1.6 on day 7; or (3) AST/ ALT > 2000 IU/L within first 7 days [9].

#### Secondary outcomes

1. Incidence of PNF
  Defined as graft loss, retransplantation, or participant death due to graft non-function in first 30 days (excluding non-function secondary to hepatic artery thrombosis, biliary complications, or recurrent hepatic disease)

2). Incidence of postoperative AKI during postoperative days 1–7
  Defined by Kidney Disease: Improving Global Outcomes (KDIGO) criteria published in 2012 [40]
3). Incidence of ARDS during postoperative days 1–7
  Defined according to Berlin modification of the American European Consensus Committee (AECC) definitions published in 2012 [42].
4). Incidence of graft failure and retransplantation rate during 3-year follow-up period.
5). All-cause mortality in the 3-year follow-up period.

### Ethics issues

#### Ethics committee

Ethical approval is obtained from the ethics committee of Renji Hospital, Shanghai Jiaotong University School of Medicine (project number: 2018–022). The principal investigator will submit a research progress report to the Ethics Committee on a regular basis.

#### Written informed consent

For every potential participant, investigators should explain in detail the purpose, procedures, and potential benefits and risks of the study in a written informed manner. They must let every potential participant know that he/she has the right to withdraw his/her consent at any time during the study period. We will inform the participants about the study as soon as possible after receiving the operation notice, though every potential participant may do not have enough time for careful consideration. Every participant or the authorized surrogate of the participant must sign the consent before they can be enrolled in the study. Written informed consents will be kept as a part of the clinical trial documents.

#### Privacy protection

Personal information of all participants will be confidentially kept. All related documents will be locked up. For each participant, all data collected during the study period will be identified by a serial number and a name acronym in the CRF. Results of the study will be published in the form of publication(s) without personal information (including name, age, etc.).

### Randomization and blinding

An independent statistician will generate a random sequence of 0 s and 1 s in a 1:1 fashion correspondent to numbers from 1 to 330. The results of allocation will be stored in the electronic system until the end of the study period.

An independent pharmacist who will not participate in the rest of the study will be noticed and also receive the results of randomization to provide the trial drugs to the participating centers in sequentially numbered, sealed, identical boxes according to the allocation sequence.

Participants who consent to enter the trial will be sequentially assigned to the random number based on the order of surgery time and receive either dexmedetomidine or placebo (normal saline).

Postoperative follow-up will be performed by investigators who do not participate in participants’ care and who have been trained prior to the study.

Both anesthesiologist(s) and investigator(s) do not communicate while collecting and analyzing results. Study personnel, healthcare team members, and participants will be blinded to the treatment group allocation throughout the study period.

### Description of study treatment

#### Premedication

No pre-anesthesia medication will be administered. Thoracic epidural anesthesia will not be sited before or after induction of anesthesia.

#### Intraoperative monitoring

Intraoperative monitoring will be instituted to include electrocardiogram (ECG), non-invasive and invasive blood pressure monitoring, pulse oximetry, central venous pressure, bispectral index (BIS), end-tidal gas analysis, nasopharyngeal temperature, and urine output. Thermodilution cardiac output monitoring (measured via a pulmonary artery catheter or PICCO system) or perioperative transesophageal echocardiography may be used when necessary.

#### Induction of anesthesia

Anesthesia will be induced with midazolam (0.015–0.3 mg/kg), remifentanil/sufentanil/ fentanyl, propofol, and rocuronium/cisatracurium. For participants with predicted difficult airway, succinylcholine may be used for rapid sequence induction. Awake fiberscope intubation may be performed. Dexamethasone (4–8 mg) may be administered to prevent postoperative nausea and vomiting.

#### Maintenance of anesthesia

1. Dexmedetomidine treatment group

Anesthesia will be maintained with sevoflurane inhalation, of which the concentration will be adjusted to maintain the BIS value between 40 and 60. Muscle relaxation will be maintained with rocuronium or cisatracurium. Analgesia will be maintained with remifentanil (administered via continuous infusion), sufentanil (administered via continuous infusion or intermittent injection), or fentanyl (administered via intermittent injection). In addition to this, participants will receive an initial loading dose of dexmedetomidine of 1 μg/kg over 10 min after the induction of anesthesia followed by a continuous infusion of 0.5 μg/kg/h until the end of surgery.

2). Control group

Participants in the placebo arm receive a corresponding amount of saline instead of DEX.

#### Intraoperative management

Mechanical ventilation will be performed with a tidal volume between 6 and 8 ml/kg, a positive end expiratory pressure of 5 cmH20, a plateau pressure of less than 30 cmH20, a frequency between 10 and 16 per minute, and a 1:1 air-oxygen mixture. Intraoperative fluid therapy will be managed according to routine practice. Packed red blood cells will be transfused to maintain hemoglobin levels within the range 7–10 g/L. Plasma, platelets, and anti-fibrinolytics will be used to manage coagulopathic states. Vasopressors/inotropes will be administered where necessary to maintain systolic blood pressure within 20% of baseline. Intravenous steroids will be administered before reperfusion of the new liver as per normal protocol.

#### Emergence from anesthesia

Towards the end of surgery, analgesia will be optimized with intravenous fentanyl/sufentanyl according to requirement. 5-HT3 antagonists may be administered for the prevention of postoperative nausea and vomiting. Sevoflurane inhalation concentration will be decreased or ceased, and muscle relaxation may not be antagonized with neostigmine or sugammadex. The treatment or control infusion will be stopped at the end of surgery. If the participant’s condition warrants a delayed emergence from anesthesia, then anesthesia will be maintained and the participant will be transferred to the intensive care unit.

#### Postoperative analgesia

Postoperative analgesia in the first 3 days will be provided with intravenous bolus administration of non-steroidal antiinflammatory drugs (flurbiprofen or coxibs, etc.) in ICU. Intravenous opioids may also be administered in participants with severe pain. PCA technique will not be recommended in post-transplantation participants in our center.

### Study procedure and data collection

#### Participant screening

The day before surgery (or Friday for participants who will undergo surgery on the next Monday), investigators who have been trained for the study and are authorized by the principal investigator will screen potential participants according to the inclusion and exclusion criteria. Participants who meet the inclusion/exclusion criteria will be invited for study participation.

#### Data collection before surgery

Baseline data will be collected after obtaining written informed consent.

1. Demographic data: gender, date of birth, height, weight, and body mass index (BMI)
2. Medical data: diagnosis (reason for LT), comorbidities, concomitant medication, non-drug therapies, history of smoking and drinking, history of food or drug allergy, and history of anesthesia and surgery
3. Results of physical examinations
4. Baseline laboratory investigations (to calculate model for end-stage liver disease [MELD] score)
5. Donor characteristics: age, height, weight, BMI, cause of death, and virology status

#### Data collection during surgery

1. Duration of surgery, type of liver grafts (cadaveric or living), operating method
2. Duration of anesthesia and doses of anesthetic drugs given
3. Fluid balance during surgery, including estimated bleeding, type and volume of blood products transfused (document duration of storage before transfusion), and type and volume of fluid infusion
4. Variations of blood pressure and BIS during anesthesia/surgery
5. Duration of cold and warm ischemia times

#### Data collection immediately after surgery

Participants will be followed up twice daily during the first 7 days following surgery and then weekly until the 30th day after surgery (by telephone for those discharged from hospital).

1. Incidence of EAD.
2. Incidence of PNF.
3. Incidence of AKI during postoperative days 1–7.
4. Incidence of ARDS during postoperative days 1–7.
5. Medication (including sedatives, analgesics, anticholinergics, and glucocorticoids) used during postoperative days 1–7 will be recorded.
6. Length of stay in ICU and hospital after surgery.
7. All-cause 30-day mortality.

#### Long-term data collection

Participants will be followed up (by telephone) at 3, 6, 12, 24, and 36 months after surgery.

1. Incidence of graft failure and retransplantation rates between 30 days and 36 months after the initial surgery
2. Status of survival. For participants who died after surgery, the date of death will be recorded.

#### End of follow-up

**Fig. 1:**
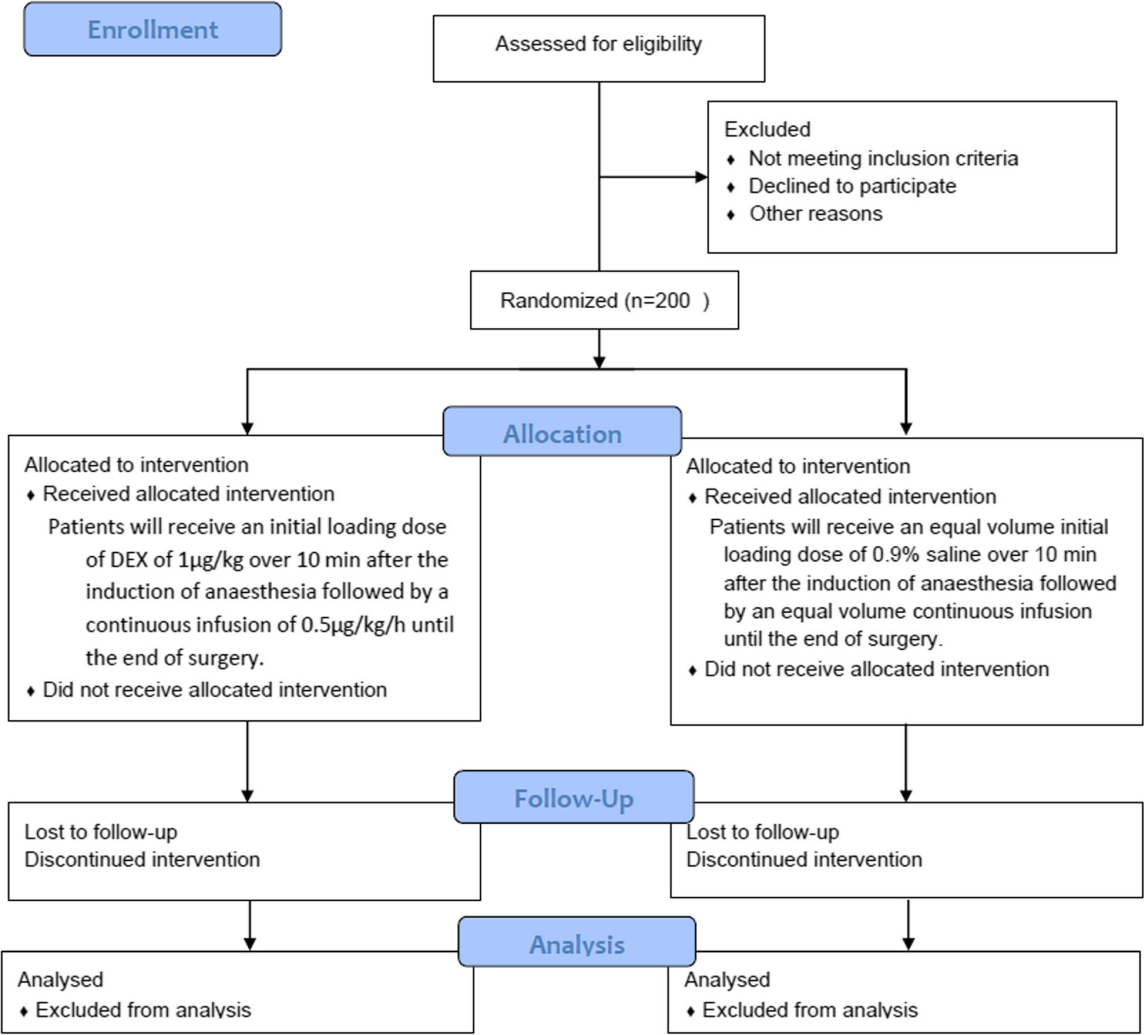
Flow chart of the trial. The present study is a randomized controlled Anesthesiology trial. Three hundred thirty LT participants will be included and randomized equally to two groups. After surgery, each participant will be followed up. Whether DEX can reduce the incidence of early postoperative EAD and PNF and even improve the postoperative survival and outcome of orthotopic liver transplantation recipients will be analyzed after data collection

**Fig. 2:**
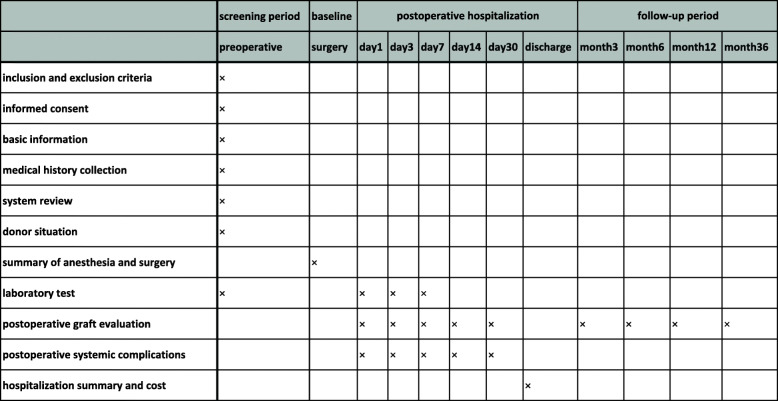
Study schedule showing time points for enrollment and assessment. The informed consent and examination will be conducted after recruitment. Then, participants will be randomized into two groups. Adverse events will be recorded in the case report form at any time during the study. Postoperative graft evaluation includes EAD, PNF, AKI, and ARDS

All participants will be followed up until the end of the 3rd year (36th month) after LT, or if they die within 3 years they will be followed up to death. The study design is illustrated in the flow chart in Fig. 1, and the study schedule is presented in Fig. 2.

### Safety considerations

In the present study, the drug under investigation is commonly used during daily anesthetic practice at a similar dose. Therefore, our study is unlikely to produce additional risk to participants. However, adverse events may occur even during routine anesthesia. In such case, anesthesiologists will manage participants according to routine practice. During the present study, the occurrence of adverse events/severe adverse events will be monitored; any adverse events/severe adverse events will be managed, followed up, and documented.

#### Adverse events

1. Possible adverse events during the study

These include anesthesia-related adverse event, general anesthesia-related adverse events, and perioperative analgesia-related adverse events.

The occurrence of adverse events will be monitored from the beginning of anesthesia until 24 h after surgery.

2). Management and documentation of adverse effects

Any adverse events should be treated promptly according to routine practice and should be followed up until it has completely resolved. Any adverse event should be documented, including time of occurrence, diagnosis, time of diagnosis, management, duration, sequelae, and severity.

The severity of adverse events can be classified into the following 3 classes:

- Mild: signs and symptoms are mild and transient, do not affect daily activity, do not need treatment, and usually recover after rest.
- Moderate: signs and symptoms last longer, mildly affect daily activity, and recover after simple treatment.
- Severe: signs and symptoms last even longer, significantly affect daily activity and life, and do not recover after simple treatment.

#### Severe adverse events

Any severe adverse events should be treated immediately according to routine practice and must be followed up until it is completely resolved or when therapy is ended. Any severe adverse event should be documented, including time of occurrence, diagnosis, time of diagnosis, management, duration of persistence, and sequelae. In case of any severe adverse event, apart from active treatment and documentation as above, the principal investigator and the Ethics Committee will be informed within 24 h in written report. In case of study drug-related death, immediately stop the clinical trial, report the event to the Ethics Committee as soon as possible, record in detail, and carefully preserve the related documents. Restart of the study will be decided by the Ethics Committee.

#### Emergency unmasking

In an emergency (e.g., rapid, unexpected deterioration in the participant’s clinical status), the anesthesiologist can request unmasking of the treatment allocation, or adjust or interrupt the study drug infusion if necessary. Likewise, the team leading postoperative care can request unmasking of treatment allocation in the interests of participant care. The detailed reasons for such events will be documented. These cases will be included in the intention-to-treat analysis.

When it is necessary to unmask, the biostatistician who generated the allocation list can be connected at any time and can open the electronic system to unmask.

### Statistics and data management

#### Statistical analysis

All analyses will be conducted based on intention-to-treat principle. Statistical analysis includes analysis of numeric variables, categorical variables, and time-to-event variables. Numeric variables including postoperative laboratory results will be presented as mean (standard deviation) or median (minimum, maximum; or interquartile range). Categorical variables including the incidence of EAD, PNF, AKI, and ARDS after LT will be presented as number of cases (percentage). Difference of numeric variables between groups will be analyzed using independent sample *t* test, and that of categorical variables will be analyzed using chi-square test. Cox proportional hazard regression will be used to estimate average effect sizes for each treatment. Results are reported as hazard ratios and 95% confidence intervals. Two-tailed tests will be used in all statistical analysis, and *p* values of less than 0.05 will be considered to be of statistical significance.

#### Sample size calculation

The sample size was calculated from the assumption that the incidence of EAD is 35% [43] and 20% of the DEX group were expected to develop EAD. One hundred thirty-six participants in each group are required to have an 80% power at the two-sided 0.05 significance level. With an anticipated 20% dropout rate, we concluded that a total of 165 participants for each group are required. After calculation, the effect size is 0.31, which is also in line with the general recommendations to detect a medium effect size.

#### Data management

Paper Case Report Form (CRF) will be used to record all data during the study. Investigators should promptly, completely, and accurately record data in the CRFs according to original observation. Supervisor(s)/study coordinator(s) will monitor the conduct of the study. The completed CRFs, after having been signed by the supervisor, will be sent to Renji Clinical Research Institute who are responsible for data entry and management. Data entry will be performed two times by two persons using the EpiData3.10 database system. A data manager will perform database check out using the SAS9.2 software. Data queries will be answered by investigators.

After data entry and database check out are completed and all problems encountered during the procedure solved, the database will be locked. The decision to lock database will be made by the principal investigator, the database manager, and a statistician who is responsible for statistical analysis.

### Quality control

Training programs will be organized for all investigators before the study. A study coordinator who is assigned by the principal investigator will be responsible to conduct the training programs and to keep the records of the training programs. Prior to the study, the investigators involved in the study will also need to complete training in the following items: the study protocol, the related standard operating procedure, and the working plan of the study; the instruction and contents of the CRF; and the considerations during conduct of the study (such as recording of adverse events and complications, etc.).

Trial-related training will be provided throughout the whole period of the study. The principal investigator and the study coordinator will organize new training program according to the progress of the study, such as training programs for new investigators, revised study protocol, or other reasons.

Supervisor(s)/study coordinator(s) will monitor the conduct of the study. The trial protocol must be strictly adhered throughout the trial period. All expected and unexpected findings will be documented promptly and accurately in order to guarantee the reliability of the values. An independent auditor at the Renji Hospital Clinical Research Institute will conduct an audit of the study.

## Discussion

The DAS-OLT trial is a long-term single-center interventional study of 330 participants aged between 18 and 65 years old undergoing liver transplantation. This study will provide characterization of this surgical population and depict whether DEX reduce the incidence of EAD on allograft cadaveric liver transplantation. Findings from this study will no doubt provide important information for anesthesia management in adult LT surgeries.

This study is special because, firstly, being one of the largest liver transplant centers in the world, our center has a mature surgical and anesthetic team that will decrease heterogeneity of medical care that participants receive. Secondly, the long time frame will help better evaluate the influence of DEX, especially on participants’ long-term survival and graft survival.

However, our study may face a number of difficulties. Because of the nature of donor liver transplantation, liver transplantation is often performed in an emergency. Therefore, the anesthesiologist performing the anesthesia is not known in advance. Knowing the infeasibility of pre-assigning an anesthesiologist for all participants, and in an effort to reduce the differences of anesthesia administration given by different anesthesiologists, we will assign 3 anesthesiologists for each included participant, and anesthesia will be conducted by anyone of them. In addition, since most of the participants are non-natives to our center, they are more likely to be followed up in their local hospitals, which may cause great difficulties to our follow-up survey. Designated staff will be responsible for giving phone calls to these participants and acquiring follow-up data. Lastly, the single-center nature impacts on the generalizability of this trial’s conclusions. A great percentage of participants undergoing LT in our institution suffer from hepatitis B infection or hepatocellular cancer, which is quite different from the scenario in other regions. In the USA, the most common indications for liver transplantation are hepatitis C virus (30%) and alcoholic liver disease (18%) [44].

The strengths of this study are as follows: first, this is a parallel-group, randomized, controlled trial which will be used for the first time to provide clinical evidence for the potential benefit of dexmedetomidine in improving outcomes of liver transplantation. Second, all laboratory tests will be performed at specific time (1, 3, 7, 14, 30 days after surgery), and the incidence of EAD, PNF, AKI, and ARDS will be determined accordingly. Third, this study will be followed up for 3 years after surgery and is long enough to observe the function of the new liver.

### Trial status

The trial is currently in the participant recruitment stage. With reference to the amount of surgery last year, the number of operations for adult liver transplantation in 2018 is more than 300, of which more than 90% met the conditions of this trial. The trial began recruitment on 14 January 2019 and is expected to complete the recruitment on 30 June 2020.

Protocol version number: DAS-OLT 3.0. Date: 31 October 2018.

## Data Availability

Supporting data are available.
